# Editorial: Applications of Fluorescence in Surgery and Interventional Diagnostics

**DOI:** 10.3389/fsurg.2021.624124

**Published:** 2021-04-12

**Authors:** Evgenii Belykh, Mark C. Preul, David L. Carr-Locke, Quyen T. Nguyen

**Affiliations:** ^1^Department of Neurosurgery, New Jersey Medical School, Rutgers University, Newark, NJ, United States; ^2^The Loyal and Edith Davis Neurosurgical Research Laboratory, Department of Neurosurgery, Barrow Neurological Institute, St. Joseph's Hospital and Medical Center, Phoenix, AZ, United States; ^3^Center for Advanced Digestive Care, Weill Cornell Medicine, New York, NY, United States; ^4^Departments of Surgery and Pharmacology, University of California, San Diego, San Diego, CA, United States

**Keywords:** fluorescence, diagnostics, optical guidance, vascular neurosurgery, microsurgery

Augmentation of the surgeon's and interventionalist's vision by advanced optical imaging, including fluorescence guidance [i.e., fluorescence-guided surgery (FGS)], is the basis for multiple innovations that will transform the workflow of all subspecialties of surgery and interventional diagnostics. Advanced optical imaging could solve multiple practical problems, making normal and abnormal tissue and cellular structures that are otherwise indistinguishable to an unaided human eye visible and making surgery and interventions for patients safer, more efficient, and successful.

In this *Frontiers* issue, “Applications of Fluorescence in Surgery and Interventional Diagnostics,” we are privileged to present a collection of 34 open-access publications that describe the frontiers in research and practice of fluorescence imaging in medicine. These articles were selected through an open peer-review process that unites experts in the field, including 220 authors and 60 reviewers and editors.

The first series of articles addresses the frontiers of wide-field fluorescence imaging in neurosurgical oncology and includes works on the major fluorophores: 5-aminolevulinic acid (5-ALA), fluorescein sodium, indocyanine green (ICG), and talaporfin sodium. 5-ALA-based imaging has seen significant growth in recent years, reflected by the high number of articles submitted for publication. Beginning with a historical review on how 5-ALA was introduced into practice (Georges et al.), we include several clinical studies on its use in various brain tumors (Goryaynov, Okhlopkov et al.), such as low-grade gliomas (Goryaynov, Widhalm et al.). The systematic analysis of a growing body of literature suggests that 5-ALA-guided surgery may impact patients' survival (Gandhi et al.).

Ways to improve fluorescence visualization through the quantification of signal intensity and its spectral signature are reviewed (Valdes et al.). Novel multispectral imaging technology based on these principles is presented for the first time in this collection (Charalampaki, Proskynitopoulos et al.). The latest clinical evidence and experience with the use of ICG is reported (Cho et al.), fluorescein sodium (Falco et al.), and talaporfin sodium (Akimoto et al.) for neurooncological applications are also presented. This subtopic concludes with a group of articles that discuss photodynamic therapy (Cramer and Chen), simulation models for fluorescence-guided brain surgery (Valli et al.), and the clinical benefits of integrating FGS in multitechnology surgical workflows (Schebesch et al.). In an invited opinion paper, Duffau discusses the role of fluorescence guidance in the surgery of malignant brain tumors in the context of the current trend for “maximal function-based resections.” It could be that the combination of both functional imaging or mapping and fluorescence techniques would be advantageous for a balanced and informed solution to maximize the goals of the surgery. Specifically, this could be achieved by first localizing the brain function through awake or asleep mapping to ensure the safety of resection. Second, localizing tumor extension through advanced optical imaging could ensure no unintentionally missed tumor tissue residuals.

Fluorescence and advance optical guidance will remain relevant and continue advancing within the surgical field as long as surgery remains a part of the neurooncology tumor management strategy. Future developments include more specific optical labels, such as fluorescently labeled peptides and nanoparticles to visualize abnormal and normal tissue better, for example, for peripheral nerves (1), or drug-free optical molecular imaging tools to visualize and discriminate normal (2) and abnormal (3) tissue.

The second series of papers reports on the advances of small-field handheld tools for open and stereotactic (Akshulakov et al.) brain tumor optical imaging, including papers on the frontiers in optical spectroscopy and RAMAN imaging (Lakomkin and Hadjipanayis), small-field confocal microscopy systems with fluorescein sodium (Belykh, Miller et al.), 5-ALA (Wei et al.), and ICG (Charalampaki, Nakamura et al.) used for contrast creation, as well as dyeless cross-polarization optical coherence tomography (Yashin et al.). These papers describe technologies for optical biopsies that are either FDA-approved or are at various stages of development. An exciting development within this realm is improving and optimizing the resection of malignant or invasive tumors by bringing a portable visualizing probe within the surgeon's hand that is a comfortable size and displays real-time *in vivo* fluorescence imaging to detect abnormal histoarchitecture. At least for brain surgery, although such technology could be used to extend the resection margins, which has correlated with increased survival, it may also be used to inform the surgeon of the tumor boundary and thus where to stop resection. These and other technologies within an operating room environment and incorporated into the surgical and pathology workflows effectively link the pathological consultation directly into the operating room. Theoretically, such technology could replace frozen section biopsies with optical biopsies, thereby increasing the yield of biopsies and the speed of surgery. Such technologies will include computer-aided image analysis (Izadyyazdanabadi et al.) and related image assessments that will guide and improve intraoperative diagnostics.

The third set of papers addresses the nuances of fluorescence imaging for vascular neurosurgery. Papers describe the evidence, techniques, and practical pearls of applying ICG contrast to augment visualization of cerebral blood flow in aneurysms (Norat et al.), cerebrovascular bypass (Cavallo et al.), and arteriovenous malformation (Foster et al.) surgeries. Advances in the design of wide-field microscopes, endoscopes, and administration for fluorescein-based angiography are described (Zhao et al.). As surgical techniques become more refined, incorporating such imaging techniques provides a view of the tissue's microvascularity with significant implications for tissue function preservation.

The fourth set of papers relays information on novel molecular fluorescent probes under development to improve FGS. Established and novel ways of imaging probe delivery to brain tumors across the blood-brain barrier are reviewed (Belykh, Shaffer et al.). Investigations of several drug-based strategies to augment already established 5-ALA-based diagnostics are reported [(a) Reinert et al.; (b) Reinert et al.]. Two papers report a new strategy of applying the drugs locally as fluorescent paint to highlight malignant cells (Kitagawa et al.; Byvaltsev et al.). Apart from oncology, a novel application of fluorescence diagnostics for clinically relevant inflammatory cell imaging is presented, which has had a significant impact on medical efficacy and the economics of treating critically ill patients with pulmonary disease (Birch et al.).

The fifth set of papers report on the frontiers of fluorescence-based techniques in visceral surgery (Ferrari-Light et al.; Wu et al.), reconstructive microsurgery (Ludolph et al.), and endoscopic diagnostics (Capuano et al.). These innovative clinical technologies include wide-field and small-field confocal imaging tools with fluorescein and ICG contrast. These fluorescence applications for surgery were among the first to be incorporated into disease diagnostics and therapeutics, such as gastrointestinal disease. They prompted the developments that launched surgical fluorescence handheld endoscopic imaging technology into other surgical disciplines, such as neurosurgery. These imaging techniques are a routine part of disease diagnostics and monitoring in many global locations, especially for precancerous and cancerous lesion management.

This constellation of basic, translational, and clinical papers will facilitate the interdisciplinary exchange of knowledge and will aid in the further progress of advanced optical-aided technologies. These authors and colleagues will lead advanced imaging efforts and champion innovations in optical navigation to improve patient outcomes and benefit healthcare systems.

## Author Contributions

All authors listed have made a substantial, direct and intellectual contribution to the work, and approved it for publication.

## Conflict of Interest

The authors declare that the research was conducted in the absence of any commercial or financial relationships that could be construed as a potential conflict of interest.
